# Deep-learning detection of mild cognitive impairment from sleep electroencephalography for patients with Parkinson’s disease

**DOI:** 10.1371/journal.pone.0286506

**Published:** 2023-08-03

**Authors:** Madan Parajuli, Amy W. Amara, Mohamed Shaban

**Affiliations:** 1 Electrical and Computer Engineering, University of South Alabama, Mobile, Alabama, United States of America; 2 Movement Disorders Center, University of Colorado, Aurora, Colorado, United States of America; Jordan University of Science and Technology, JORDAN

## Abstract

Parkinson’s disease which is the second most prevalent neurodegenerative disorder in the United States is a serious and complex disease that may progress to mild cognitive impairment and dementia. The early detection of the mild cognitive impairment and the identification of its biomarkers is crucial to support neurologists in monitoring the progression of the disease and allow an early initiation of effective therapeutic treatments that will improve the quality of life for the patients. In this paper, we propose the first deep-learning based approaches to detect mild cognitive impairment in the sleep Electroencephalography for patients with Parkinson’s disease and further identify the discriminative features of the disease. The proposed frameworks start by segmenting the sleep Electroencephalography time series into three sleep stages (i.e., two non-rapid eye movement sleep-stages and one rapid eye movement sleep stage), further transforming the segmented signals in the time-frequency domain using the continuous wavelet transform and the variational mode decomposition and finally applying novel convolutional neural networks on the time-frequency representations. The gradient-weighted class activation mapping was also used to visualize the features based on which the proposed deep-learning approaches reached an accurate prediction of mild cognitive impairment in Parkinson’s disease. The proposed variational mode decomposition-based model offered a superior accuracy, sensitivity, specificity, area under curve, and quadratic weighted Kappa score, all above 99% as compared with the continuous wavelet transform-based model (that achieved a performance that is almost above 92%) in differentiating mild cognitive impairment from normal cognition in sleep Electroencephalography for patients with Parkinson’s disease. In addition, the features attributed to the mild cognitive impairment in Parkinson’s disease were demonstrated by changes in the middle and high frequency variational mode decomposition components across the three sleep-stages. The use of the proposed model on the time-frequency representation of the sleep Electroencephalography signals will provide a promising and precise computer-aided diagnostic tool for detecting mild cognitive impairment and hence, monitoring the progression of Parkinson’s disease.

## 1. Introduction

Parkinson’s Disease (PD) [1, 2] is a complex neurodegenerative disease that is characterized by motor symptoms such as: slowness of movement and tremor as well as non-motor symptoms including cognitive and memory changes, anxiety, depression and sleep problems. According to Parkinson’s foundation, 1 million patients were diagnosed with the disease in the U.S. and 10 million individuals suffer from the disease worldwide [3].

The disease is depicted as challenging for physicians and specialists to diagnose and grade. Observation of motor system abnormalities is the current means of clinical diagnosis despite being subjective and prone to human error. It was also reported in [4] that the accuracy of the clinical diagnosis performed by movement disorders experts is unsatisfactory (79.6% initial assessment accuracy and 83.9% follow-up assessment accuracy). Hence, earlier and precise detection of PD and initiation of neuroprotective treatments are crucial to improve the disease prognosis and possibly slow down its progression.

Several state-of-the-art deep-learning techniques have been recently proposed for PD diagnosis, staging and biomarkers detection based on Electroencephalography (EEG), Magnetic Resonance Imaging (MRI), speech tests, handwriting exams and sensory data [5]. It was reported in [5] that the majority of the deep-learning techniques exploit either resting state EEG or handwriting/sensory data. As compared to CT, PET and MRI, EEG is relatively an inexpensive tool used for the diagnosis of several brain diseases including epilepsy, tumors and stroke. In addition, EEG derived from polysomnography is the gold standard measure for evaluation of sleep. Even if imaging modalities such as CT, MRI or PET were attempted during sleep, EEG would still be required to distinguish between sleep and wake and to distinguish between sleep stages.

There have been several studies that have shown that subjects with PD exhibit unique EEG biomarkers including decreased *β* (12–35 Hz) and γ (> 35 Hz) powers [6, 7], slowing of resting-state oscillatory brain activity [8, 9] and significant changes in phase-amplitude coupling when compared to healthy controls (HC) [10, 11]. Patients with PD frequently experience sleep disorders, including insomnia, rapid eye movement (REM) sleep behavior disorder (RBD), and excessive daytime sleepiness [12, 13]. In addition, PD is characterized by alterations in sleep architecture, including reductions in REM sleep which plays a vital role in consolidating procedural memory and motor skills [14]. Recent studies have shown that both REM and non-REM (NREM) sleep exhibit unique features in PD and PD with dementia as compared to healthy controls (HC), including lower stability, higher slowing ratio, an increase in spectral power in the *δ* (1–4 Hz) and *θ* (4–8 Hz) bands during REM, as well as lower baseline power in *σ* waves (12–15 Hz) within the parietal regions during the NREM sleep stages [15–19]. Since patients with PD have both cognitive and motor dysfunction, alterations in sleep stages have potential clinical implications.

Mild cognitive impairment (MCI) which is a non-motor complication of PD can be visual, spatial dysfunction, or executive dysfunction, that may occur with or without memory loss. In addition, cognitive impairment has been related to disease morbidity, significant burden on caregivers, social and working impairment, placement at long-term care facilities, and mortality [20]. There are currently no established biomarkers or effective treatments for cognitive impairment, but earlier identification of impairment may allow earlier intervention which is urgently needed to improve the prognosis of the disease.

Machine and Deep Learning (MDL) techniques [21–29] have been shown to successfully screen patients based on dataset modalities including MRI [30–32], speech patterns [33–38], sensory or handwriting data [39–51] and EEG [52–72]. However, the use of MDL for the detection of PD-MCI or the prediction of the risk of progression of PD to MCI and the identification of the disease biomarkers in sleep EEG have not yet been addressed.

In this paper, we introduce two novel deep-learning based frameworks to screen and classify subjects into PD patients with normal cognition (NC) or with MCI at a high accuracy up to 99.9%. In both frameworks, a convolutional neural network (CNN) was applied on the time-frequency representation (TFR) of each sleep stage of recorded and verified EEG signals for PD patients. The contributions of the proposed work can be summarized as follows:

1. To the best of our knowledge, this is the first work to address the use of deep learning for the detection of MCI in sleep EEG for patients with PD.
2. The proposed frameworks are capable of screening patients at a significantly high 4-fold cross validation accuracy, sensitivity, specificity, quadratic weighted Kappa score and area under curve (AUC).
3. The feature maps of the last convolutional layer of the highest-performing deep-learning framework have been identified using the Grad-CAM method [79] to highlight the spatiotemporal features of MCI during the different sleep-stages.

Finally, we would also like to emphasize that we have used the term (Normal Cognition or NC) throughout the paper to refer to PD patients without any cognitive dysfunction including MCI or dementia. However, the term (Healthy Controls or HC) only refers to subjects with no decisive clinical diagnosis of PD and those subjects were not considered in this study. The remainder of this paper is organized as follows. Section 2 provides an overview on the related work. Section 3 presents the dataset adopted in this study, followed by a detailed description of the proposed approach in Section 4. The experimental study and results are then discussed in Section 5. Finally, the conclusion and discussion are presented in Section 6.

## 2. Related work

Vanegas *et al*. used extra tree, logistic regression and decision tree to identify the EEG based biomarkers of PD achieving Area Under Curve (AUC) of the Receiver Operating Characteristic (ROC) curve of 99.4%, 94.9% and 86.2% [52]. The models were applied on the EEG spectral amplitudes of the posterior occipital area of the brain during visual stimulation. The weights of the logistic regression along with the decision nodes of the decision tree were used to identify the frequencies that discriminate the PD subjects from HC. The theta, alpha to beta frequency ranges were found to be the most influential frequencies in the classification problem.

Oh *et al*. developed a 13-Layer Convolutional Neural Network (CNN) in order to classify subjects into PD and controls using a resting-state EEG for 20 PD subjects and 20 HC [53]. The model achieved an accuracy of 88.3%, a sensitivity of 84.7% and a specificity of 92%. Wagh *et al*. introduced an 8-layer graph CNN applied on EEG features for 1,385 subjects with neurological diseases including PD and 208 HC achieving an AUC of 85% in detecting the diseases [54].

Koch *et al*. used a machine learning model based upon Random Forest in order to classify PD subjects into patients with good or poor cognition. The model was applied on clinical and automated EEG features achieving an AUC of 91% AUC [55]. Shi *et al*. developed two hybrid models based upon two dimensional and three dimensional CNN and Recurrent Neural Network (RNN) frameworks where the three dimensional CNN-RNN achieved an accuracy of 82.89% outperforming the two dimensional model [56]. Lee *et al*. have also introduced a hybrid model based on CNN and Long Short Term Memory (LSTM) in order to classify subjects into PD and HC at an accuracy of 96.9% [57].

Khare *et al*. have used several machine learning methods including the Least Squares Support Vector Machine (LSSVM) on features that have been identified from the tunable Q-factor wavelet transform (TQWT) applied on resting-state EEG dataset related to 15 PD subjects and 16 HC [58]. The LSSVM model was able to differentiate between HC and PD subjects with and without medications at an accuracy of 96% and 97.7% respectively. In addition, Khare *et al*. have also developed a 10-Layer CNN that was applied on the smoothed pseudo-Wigner Ville distribution (SPWVD) transformation of two EEG datasets representing 35 PD subjects and 36 HC [59]. The model achieved an accuracy of 99.9% and 100% on the two datasets respectively. Loh *et al*. have applied a 2D-CNN on the Gabor transform of a resting-state EEG dataset of 15 PD subjects and 16 controls in order to classify subjects into HC and PD with and without medications at an accuracy of 99.5% [60].

Shaban has also developed three 13-layer ANN models applied on the Oz, P8, and FC2 channels of a resting state EEG dataset for 15 PD subjects and 16 HC [61]. The models outputs were fused using a majority voting method achieving an accuracy of 98% for classifying subjects into PD or HC. In addition, Shaban *et al*. have recently developed a Continuous Wavelet Transform (CWT) based CNN where a 20-layer CNN model was trained and validated on the Morlet wavelet transform [62, 63] and the second order derivative of the wavelet transform [64] of a resting-state EEG at an accuracy up to 99.9% for distinguishing PD patients from HC.

In [65], Khare *et al*. introduced the use of the automated extreme learning machine (AOELM) algorithm on the automated variational mode decomposition (AOVMD) of a resting-state EEG dataset related to 16 HC and 15 PD subjects. The model was capable of classifying the subjects into PD off medication and HC at an accuracy of 98.9% and distinguishing PD on medication from HC at an accuracy of 98.6%. Further, in [66], Chawla *et al*. introduced a machine-learning framework using the k-nearest neighbors (KNN), SVM, logistic, and radial basis function (RBF) classifiers to differentiate PD subjects from HC. The classifiers were applied on a set of entropy features extracted from the flexible analytic wavelet transform (FAWT) of two resting-state EEG datasets. The proposed framework based upon KNN was able to classify subjects at an accuracy of 99% and 95.9% based on the two datasets respectively. Table 1 shows a summary of the-state-of-the-art machine and deep-learning methods used for PD diagnosis, staging and biomarkers identification based upon awake EEG.

**Table 1:** The state-of-the-art machine and deep-learning techniques applied on awake EEG for PD diagnosis, staging and biomarkers identification.

Reference	Application	Dataset Size	Method	Performance
Vanegas *et al*. [52]	PD Biomarkers Extraction	29 PD and 30 HC	Extra Tree, Logistic Regression, Decision Tree	AUC: 99.4%, 94.9%, 86.2%
Oh *et al*. [53]	PD Diagnosis	20 PD and 20 HC	13-Layer CNN	Accuracy: 88.25%
Wagh *et al*. [54]	Diagnosis of Several Neurological Diseases	1,385 Diseased and 208 HC	8-Layer Graph CNN	AUC: 0.9
Koch *et al*. [55]	Cognition Level Classification for PD	20 Good Cognition and 20 Poor Cognition	Random Forest	AUC: 91%
Shi *et al*. [56]	PD Diagnosis	40 PD and 30 HC	Two and Three- dimensional CNN-RNN	Accuracy: 81%, 83%
Lee *et al*. [57]	PD Diagnosis	20 PD and 22 HC	CNN-LSTM	Accuracy: 97%
Khare *et al*. [58, 59]	PD Diagnosis	35 PD and 36 HC	Tunable Q-factor Based LSSVM, SPWVD Based CNN	Accuracy: 97.7%, 99.5%
Loh *et al*. [60]	PD Diagnosis	15 PD and 16 HC	Gabor-Transform Based 8-Layer CNN	Accuracy: 99.5%
Shaban *et al*. [61–63]	PD Diagnosis	15 PD and 16 HC	13-Layer ANN, Wavelet-Based 12-Layer CNN	Accuracy: 98%, 99.9%, 99.9%
Khare *et al*. [65]	PD Diagnosis	15 PD and 16 HC	AOVMD-Based AOELM	Accuracy: 98.9%, 98.6%
Chawla *et al*. [66]	PD Diagnosis	35 PD and 36 HC	FAWT-Based KNN	Accuracy: 99%, 95.9%

In addition to the use of machine and deep-learning for PD diagnosis, staging and biomarkers identification based on EEG data, various studies have used non-machine learning techniques to detect PD as well as investigate the influence of EEG electrodes number and location on the models’ performance. Sahota *et al*. proposed a novel representation for the EEG time-series data by transforming a single EEG time-series into a 7-variate series of coefficients per 20s or 30s epoch of data reducing the sampling rate of the data from 256Hz to 0.05Hz [67]. Further, MrSQL time-series classification method was applied on the transformed data to differentiate PD from HC achieving an accuracy of 90.2% based on wakeful EEG data and up to 95.5% on N3 and REM sleep EEG data. Suuronen *et al*. investigated the significance of the number and the location of the EEG device electrodes on the classification accuracy of machine learning algorithms used to differentiate PD from HC based on resting-state EEG data [68]. Using a budget search algorithm, it was shown that the performance of a logistic regression classifier measured using a nested 10-fold cross validation improved when patients’ eyes were open rather than closed. Based on the eyes open data, it was also demonstrated that the best performance was obtained where AUC > 0.7 using only five channels placed far away from each other.

Several other studies have experimented the use of deep-learning on raw EEG recordings for the automatic detection of Schizophrenia at an accuracy of up to 97% using a fine-tuned short-time Fourier transform based VGG-16 [69] and an accuracy up to 99.5% using a CWT based VGG-16 [70]. Further, a fine-tuned pre-trained VGG-16 model was introduced to classify the Hilbert spectrum images of the first four intrinsic mode functions (IMFs) components obtained by applying the Empirical Mode Decomposition on the EEG recordings for patients with schizophrenia and HC [71]. The deep-learning model was able to obtain an accuracy of up to 98.2% in differentiating Schizophrenia from HC. Other studies proposed using a set of pre-trained and fine-tuned deep-learning models including VGG-16, DenseNet121, ResNet101 and Xception models for the automated detection of migraine from the CWT images of EEG signals for 39 subjects (i.e., 18 migraine patients and 21 HC) [72]. A maximum accuracy of 100% was achieved using the VGG-16 model for classifying subjects into patients with migraine and HC.

Although several methods have been proposed for the detection and staging of PD based upon awake EEG, the use of deep-learning for identifying the cognitive complications of PD including MCI or dementia based upon sleep EEG has not yet been investigated. To address this critical issue, we propose a TFR based deep-learning framework that exploits the sleep stages of EEG that were recorded for patients with PD in order to classify the subjects into patients with NC or MCI as well as visualize the features attributed to MCI.

## 3. Materials

This study leveraged a previously collected dataset including 36 participants with idiopathic PD. PD participants (age: 65.5 ± 7.1; 62.7% male; race: Caucasian (91.5%) and African American (8.5%) participants; duration of PD: 5.63 ± 4.45 years; years of education: 15.7 ± 2.47 years) were recruited from the University of Alabama at Birmingham (UAB) Movement Disorders Center.

Inclusion required age ≥ 45 years, clinical diagnosis of idiopathic PD, Hoehn and Yahr stage 2–3 and stable medication regimen for at least 4 weeks prior to study entry. Exclusion criteria included examination or historical findings suggestive of secondary or atypical Parkinsonism, Montreal Cognitive Assessment (MoCA) score < 18, untreated sleep apnea, or use of investigational drugs.

All PD participants underwent overnight laboratory-based polysomnography, with study starting at approximately 10 pm and the duration of recording was 8 hours. EEG was recorded using 6 electrodes (i.e., F3, F4, C3, C4, O1, O2) at a sampling rate of 512 S/s. Both Electromyography (EMG) and Electrooculography (EOG) data was collected as well.

Additionally, all participants were evaluated with a level II neurocognitive battery as defined by the PD-mild cognitive impairment diagnostic criteria as recommended by the Movement Disorders Society (MDS), with at least two tests in each of five cognitive domains [73]. Classification of PD-MCI or PD-NC was determined using this assessment with 16 subjects having MCI and 20 subjects having NC. All PD participants were also evaluated with the MDS-Unified PD Rating Scale (UPDRS).

In addition, for each of the subjects that have been identified as PD patients with NC or MCI, a label was assigned to each fixed time interval (i.e., 30 seconds) of the sleep EEG time-series in order to refer to the corresponding sleep stage (i.e., N1, N2, N3 and REM). In this study, we have mainly focused on N2, N3 and REM only since the N1 sleep stage is light sleep and often combined with α frequencies (8–12 Hz) of wakefulness.

## 4. Methods

Prior to the use of the dataset in this study, the institutional review boards for both the University of South Alabama and the University of Alabama at Birmingham have determined that this study is not subject to FDA regulations and is not considered human subjects research. De-identified subjects’ data were shared between the two institutions and a written informed consent was obtained from all participants.

**Fig 1:**
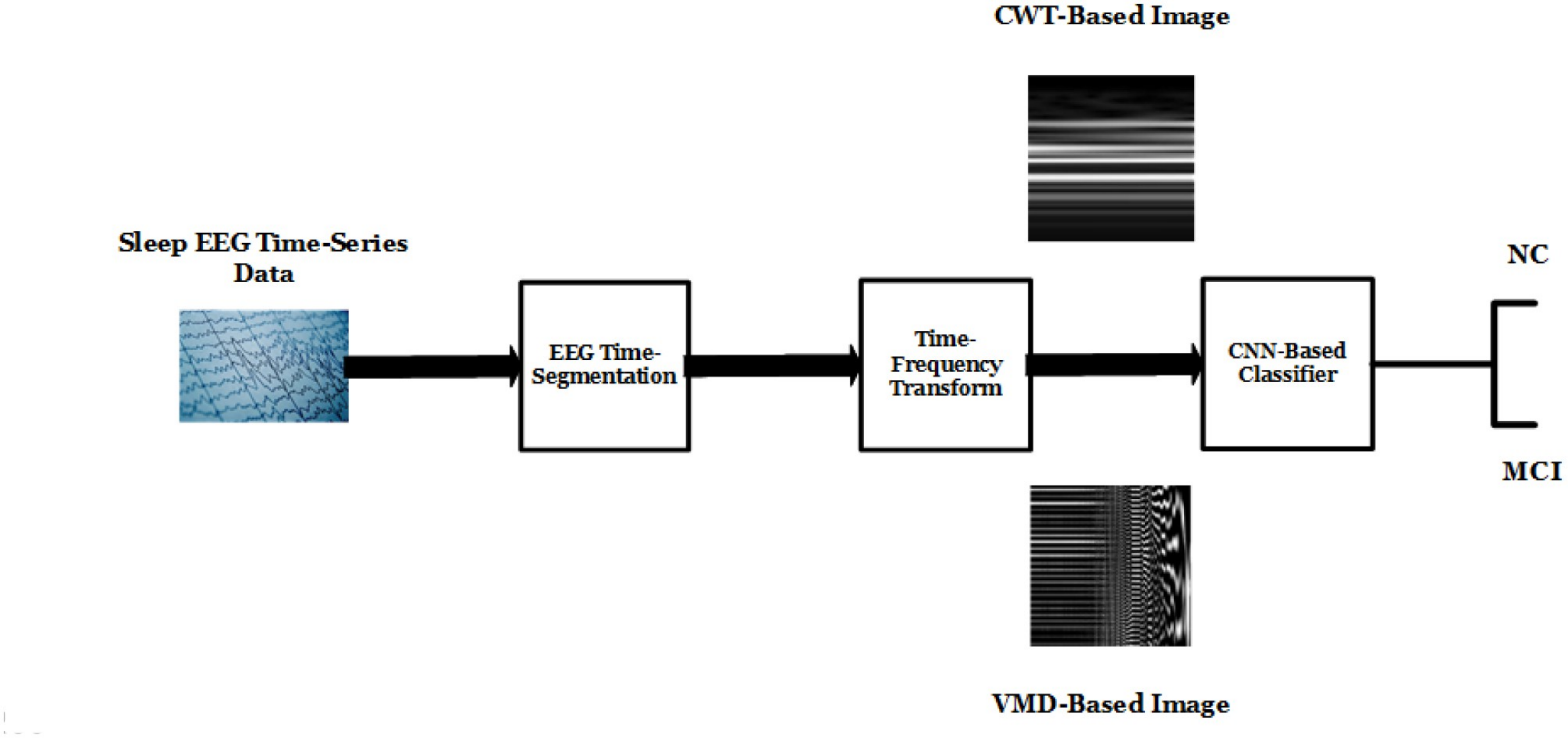
Proposed deep-learning framework.

In this section, we introduce two deep-learning based frameworks applied on the TFR representation of each sleep-stage segment of the sleep EEG time-series. The proposed frameworks consist of three main stages: sleep-stage extraction, TFR, feature selection and classification using proposed CNN models. The three stages of the proposed framework (See Fig 1) are described as follows:

### 4.1 Sleep-stage extraction

Each EEG time-series recorded for each subject mainly represents the four sleep-stages. Excluding N1 from our analysis, each of the N2, N3 and REM sleep-stage EEG signals were extracted for each subject and at each channel using the manually provided expert-based labels.

### 4.2 TFR

Two TFR representation approaches were considered in this study; the CWT [74] and the variational mode decomposition (VMD) [75].

Using the CWT method, each sleep-stage EEG time-series was transformed into a two-dimensional matrix *X*_*i*_ with each value defined as follows:

$$X_{i}\left(s,T\right)=\frac{1}{\sqrt{s}}\int_{0}^{\infty }x_{i}(t)\varphi (\frac{t-T}{s})dt$$ (1)

where *x*_*i*_*(t)* is the sleep-stage EEG time-series measured at the *i*^th^ electrode, *φ* is the Morlet analysis wavelet, T is the translation and *s* is the scale of the Wavelet where the scale is the reciprocal of the Fourier frequency. As shown from Eq 1, the transformed signal *X*_*i*_ (*s*,*T*) is a function of both time and scale.

Each *X*_*i*_ matrix was calculated using Eq 1 for each sleep stage EEG signal captured at each electrode and related to each subject. We found that the matrix has 146 rows for N3 or REM EEG signals and 176 rows for N2 signals where each row represents a scale. The bottom rows represent lower scale values whereas the top rows represent higher scale values. This may show that the N2 signals are composed of more scales or Fourier frequency components with respect to other sleep stage signals.

**Fig 2:**
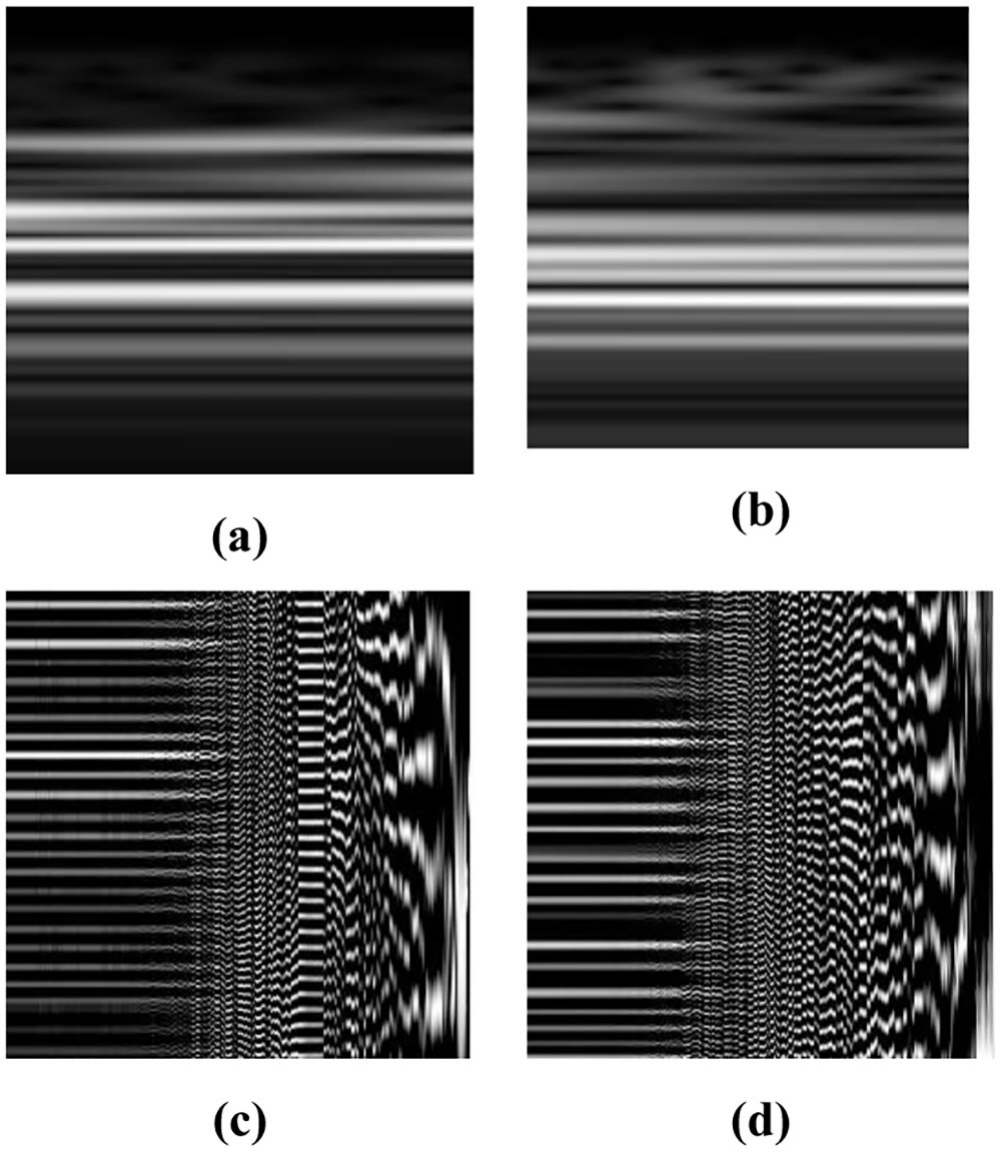
(a) N3-CWT for PD-NC (b) N3-CWT for PD-MCI (c) N3-VMD for PD-NC (d) N3-VMD for PD-MCI.

Due to the large number of columns in each *X*_*i*_ matrix representing the total number of time-samples recorded during an 8-hour sleep and to reduce the complexity of the following deep-learning methods, the *X*_*i*_ matrices were further segmented across the columns creating 146×146 (N3/REM) or 176×176 (N2) smaller matrices. The absolute values of the 146×146 (N3/REM) or 176×176 (N2) matrices were calculated and normalized (i.e., were divided by the maximum absolute value of each matrix). The normalized matrices were then provided to the following proposed CNN-based model. Fig 2(a) and 2(b) shows the N3-CWT images for PD subjects with NC and MCI respectively.

Using the VMD method, each sleep-stage EEG signal was decomposed and simplified into a finite number of components or IMFs, each related to an independent source or a frequency. The objective of VMD is to minimize the bandwidth of each mode such that the sum of all the modes are equivalent to the original EEG sleep-stage signal. As a result, the VMD method is noise-resilient, ensuring an almost accurate separation of the components of the EEG signal. The VMD optimization method is defined as follows:

$${min}_{B_{n}}\sum_{n}\left\|\frac{d}{dt}\left[\left\{\left(\delta \left(t\right)+\frac{j}{\pi t}\right)*s_{n}(t)\right\}e^{-jB_{n}t}\right]\right\|$$ (2)

$$s.t.\;\sum_{n}s_{n}\left(t\right)=f\left(t\right)$$

where *f(t)*, *s*_*n*_*(t)* and *B*_*n*_ represent the sleep-stage EEG time-series, the IMFs of the signal *f(t)* and the bandwidths of the IMFs respectively. The IMFs of each sleep-stage EEG signal for each subject and at each electrode were then calculated using Eq 2 and represented as two-dimensional matrices with the columns of each matrix representing the IMFs of the signal while the rows represent the time-index. The left-most columns correspond to lower-frequency modes whereas the right-most columns represent higher-frequency modes. The number of modes was arbitrarily selected (e.g., 256) to ensure the simplicity of the required computations to execute the constrained optimization approach without losing any significant components of the original sleep-stage EEG signals.

Further, each VMD matrix was further segmented across the rows generating square matrices of 256×256 values. Each square matrix was then normalized, resized to 512×512, and finally provided as an input to the following proposed CNN model. Fig 2(c) and 2(d) shows the N3-VMD images for PD subjects with NC and MCI respectively.

### 4.3 Feature extraction and classification using the proposed CNNs

**Fig 3:**
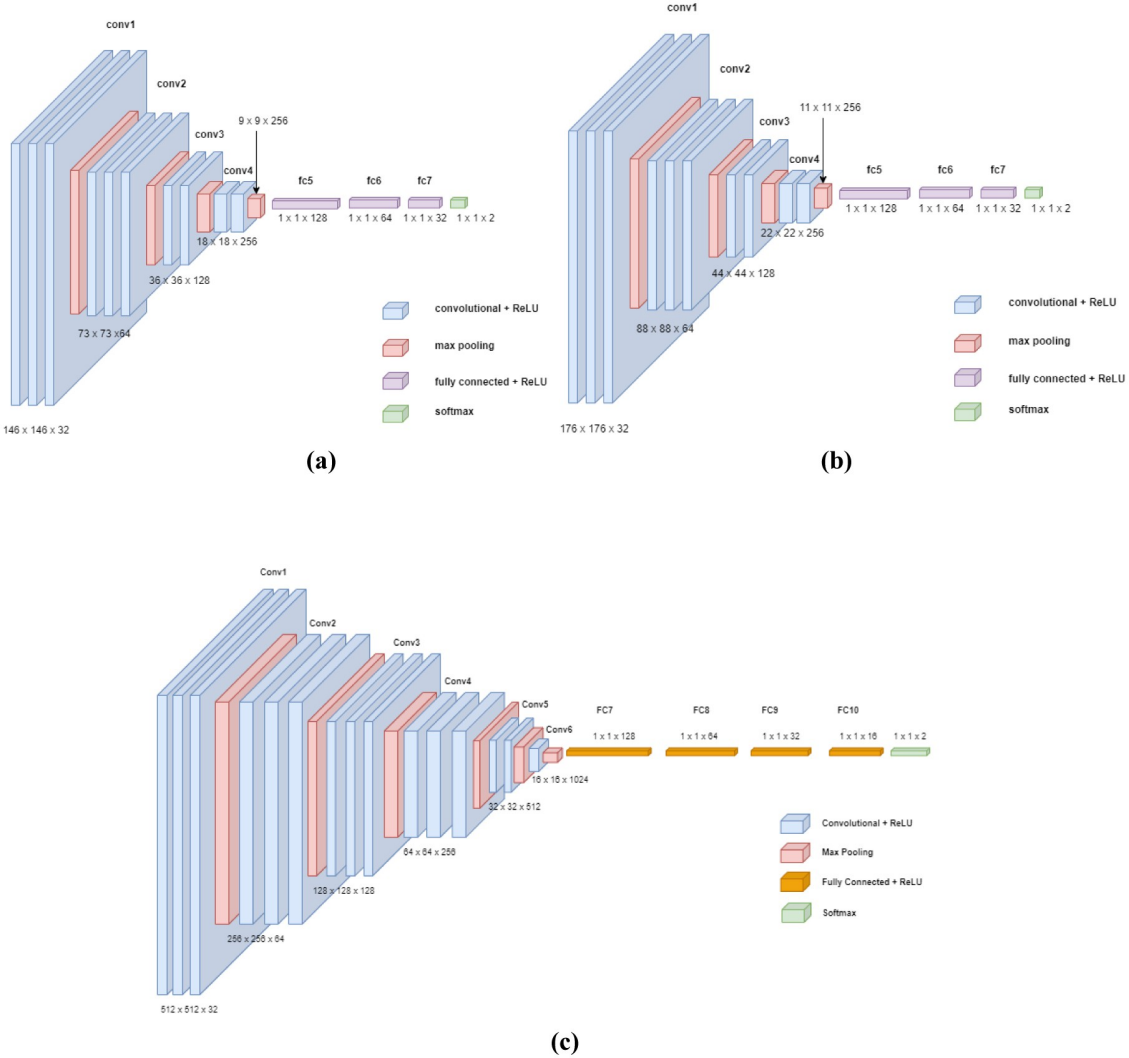
Proposed CNN for (a) N3 and REM CWT data (b) N2 CWT data (c) VMD data.

The proposed CNN models applied on the CWT and VMD data are described in Fig 3.

The proposed CNN models were trained and 4-fold cross validated in order to classify subjects into PD with NC or PD with MCI. The proposed CNNs layers description is detailed in Tables 2 and 3 when the CNNs were applied on both CWT and VMD data respectively.

**Table 2:** Components of the proposed CNN model applied on the CWT data.

Layer	No. of Layers	Layer Size	No. of Kernels	Kernel Features
Input Layer	1	176 × 176 × 3 (N2)/ 146 × 146 × 3 (N3/REM)	-	-
Convolution/ReLU	3	3 × 3	32	Same Padding
Pooling Layer	1	2 × 2	32	No Padding
Convolution/ReLU	3	3 × 3	64	Same Padding
Pooling Layer	1	2 × 2	64	No Padding
Convolution/ReLU	2	3 × 3	128	Same Padding
Pooling Layer	1	2 × 2	128	No Padding
Convolution/ReLU	2	3 × 3	256	Same Padding
Pooling Layer	1	2 × 2	256	No Padding
Fully Connected/ ReLU	3	128, 64, 32	1	-
Fully Connected	1	2	1	-
Softmax/Classification	1		1	-

**Table 3:** Components of the proposed CNN model applied on the VMD data.

Layer	No. of Layers	Layer Size	No. of Kernels	Kernel Features
Input Layer	1	512 × 512 × 3	-	-
Convolution/ReLU	3	3 × 3	32	Same Padding
Pooling Layer	1	2 × 2	32	No Padding
Convolution/ReLU	3	3 × 3	64	Same Padding
Pooling Layer	1	2 × 2	64	No Padding
Convolution/ReLU	3	3 × 3	128	Same Padding
Pooling Layer	1	2 × 2	128	No Padding
Convolution/ReLU	3	3 × 3	256	Same Padding
Pooling Layer	1	2 × 2	256	No Padding
Convolution/ReLU	2	3 × 3	512	Same Padding
Pooling Layer	1	2 × 2	512	No Padding
Convolution/ReLU	1	3 × 3	1024	Same Padding
Pooling Layer	1	2 × 2	1024	No Padding
Fully Connected/ ReLU	3	128, 64, 32,16	1	-
Fully Connected	1	2	1	-
Softmax/Classification	1		1	-

## 5. Results

In this study, we aimed at studying N3 at both frontal channels (F3, and F4) and analyzing both N2 and REM at central channels (C3, and C4). This is because delta frequencies in N3 are most prominent in the frontal regions and that is associated with increased brain metabolic activity in the frontal lobes [76]. Further, sleep spindles in N2 are generated in the thalamus and are most easily detected in the central leads in a sleep study [77]. REM sleep can be detected well in any leads, but the rapid eye movements are sometimes detected in the frontal leads as artifacts.

### 5.1 Performance of the proposed CWT-based CNN

Following the sleep-stage EEG time-series extraction, time-segmentation, and CWT application, we have obtained a number of segments per each sleep-stage as indicated in Table 4.

**Table 4:** CWT dataset description.

Sleep Stage	Training Data Size
**N2**	1,332,741
**N3**	406,886
**REM**	415,171

Based on the sleep EEG data collected, we were able to obtain a number of N2 CWT segments that are almost three times the number of samples obtained for N3 or REM. This is due to the fact that N2 is considered the longest period representing the deeper sleep. The two dimensional CWT segments were then provided to the proposed CWT-based CNN with training parameters defined in Table 5 that were identified by varying the number of epochs, the optimizer, the learning rate and the batch size using the grid search algorithm [78] while monitoring the 4-fold cross validation performance of the model (i.e., 4-fold cross-validation accuracy, sensitivity, specificity, AUC and quadratic weighted Kappa (QWK) score).

**Table 5:** Proposed CNN training parameters.

No. of Epochs	100
**Optimizer**	Adam Optimizer
**Learning Rate**	0.0001
**Batch Size**	32

For each sleep-stage, the 4-fold cross validation performance of the model was reported in Table 6. It is clear from Table 6 that the proposed model achieved an accuracy, sensitivity and specificity within the range of 92% to 96%, 91% to 95% and 91% to 95% respectively across the three sleep-stages. It was also observed that the model offered a relatively higher accuracy up to 95.9% and elevated QWK of 0.91 when applied on the N3 sleep-stage signals as compared with N2 or REM signals. This may indicate that N3 may exhibit discriminative features of PD-MCI and PD-NC. Further, it was also shown that the CWT-based CNN achieved the highest sensitivity of 94.9% and specificity of 95% for detecting PD-MCI when applied on N3 (F3). These preliminary findings motivate the need for further future clinical analysis of the significance of and relevance of N3 sleep stage EEG particularly recorded at the frontal regions of the brain to the PD-MCI early diagnosis and detection.

**Table 6:** Performance of the proposed CWT-based CNN.

	Accuracy	Sensitivity	Specificity	AUC	QWK
**N2 (C3)**	92.1% ± 0.6%	91.2% ± 0.6%	91.5% ± 0.5%	0.97 ± 0.002	0.83 ± 0.012
**N2 (C4)**	93.1% ± 0.7%	92.6% ± 1%	93.2% ± 0.8%	0.98 ± 0.003	0.87 ± 0.014
**N3 (F3)**	95.8% ± 0.5%	94.9% ± 0.4%	95% ± 0.6%	0.98 ± 0.002	0.90 ± 0.01
**N3 (F4)**	95.9% ± 0.5%	94.7% ± 0.4%	94.9% ± 0.4%	0.98 ± 0.004	0.91 ± 0.011
**REM (C3)**	92.9% ± 0.5%	91.9% ± 0.4%	92.3% ± 0.4%	0.97 ± 0.003	0.85 ± 0.009
**REM (C4)**	94.7% ± 0.9%	93.9% ± 1%	94.1% ± 1%	0.98 ± 0.006	0.88 ± 0.02

**Fig 4:**
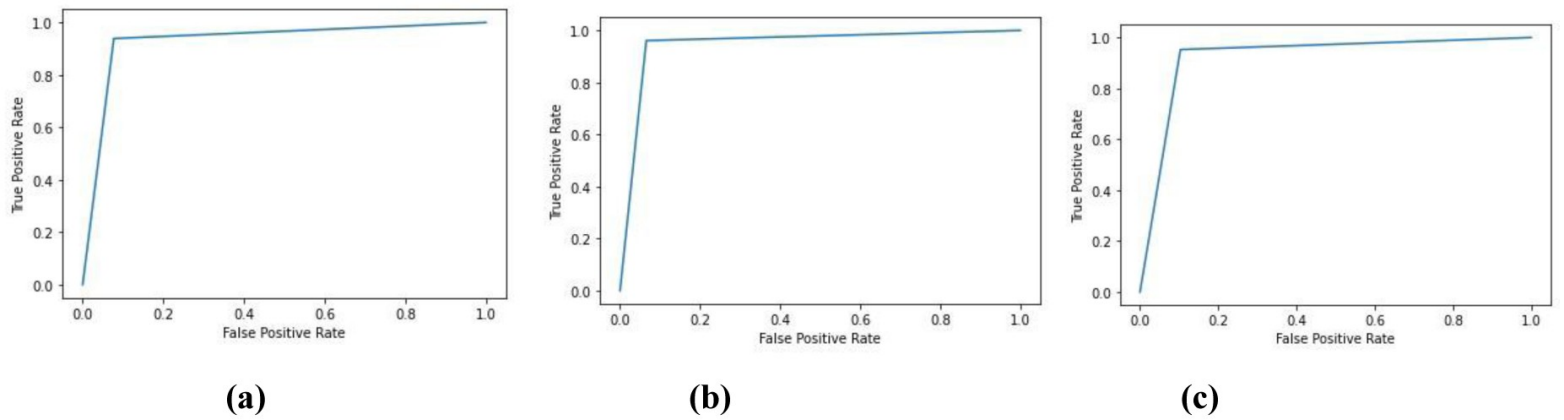
ROC of the proposed classifier applied on (a) N2 (C3) (b) N3 (F3) (c) REM (C3).

Further, Fig 4 shows the receiver operating characteristic (ROC) curves of the proposed model which appear to be almost identical across the three sleep-stages. It is also obvious that the model exhibits high separability between the two classes of interest (i.e., MCI and NC) with a true positive rate of 1 at a false positive rate that is nearly less than 0.1. From the ROC curve for the model applied on N3 EEG at the F3 shown in Fig 4(b), the model exhibits a slightly improved AUC as compared to the same model when applied on N2 as well as REM EEG data.

Further, the confusion matrix for the worst-performing CWT-based CNN when applied on the testing set of the N2 (C3) data is provided in Table 7. The rows represent the ground truth while columns represent the predictions.

**Table 7:** Confusion matrix of the proposed CWT-based CNN on N2 (C3).

	NC	MCI
**NC**	160,415	14,903
**MCI**	13,893	143,973

It is clear from Table 7 that both the true positive and true negative image counts are significantly high as compared to the false positive and false negative counts. This proves the ability of the CWT-based CNN classifier to successfully identify NC or MCI at a high rate.

### 5.2 Performance of the proposed VMD-based CNN

Once again, we have obtained a number of VMD-based samples as indicated in Table 8 following the segmentation of each sleep-stage EEG signal and the application VMD constrained optimization.

**Table 8:** VMD dataset description.

Sleep Stage	Training Data Size
**N2**	303,550
**N3**	232,055
**REM**	236,779

The proposed CNN was then trained and a 4-fold cross-validated on the VMD-based segments with the model training parameters presented in Table 9 was conducted. The optimized hyperparameters shown in Table 9 were obtained using the grid search algorithm [62].

**Table 9:** Model training parameters.

No. of Epochs	25
**Optimizer**	Adam Optimizer
**Learning Rate**	0.00001
**Batch Size**	8

The accuracy, sensitivity, specificity, AUC and QWK were also reported for each sleep-stage (see Table 10).

**Table 10:** Performance of the proposed VMD-based CNN.

	Accuracy	Sensitivity	Specificity	AUC	QWK
**N2 (C3)**	99.2% ± 0.8%	98.5% ± 0.9%	98.7% ± 1%	0.99 ± 0.002	0.98 ± 0.02
**N2 (C4)**	99.4% ± 0.6%	98.7% ± 1.2%	99% ± 0.8%	0.99 ± 0.0007	0.98 ± 0.016
**N3 (F3)**	99.8% ± 0.1%	99.2% ± 0.5%	99.4% ± 0.5%	0.99 ± 0.0004	0.99 ± 0.007
**N3 (F4)**	99.6% ± 0.4%	99.5% ± 0.5%	99.6% ± 0.4%	0.99 ± 0.0001	0.99 ± 0.009
**REM (C3)**	99.5% ± 0.4%	99.1% ± 0.6%	99.3% ± 0.6%	0.99 ± 0.0001	0.98 ± 0.008
**REM (C4)**	99% ± 1%	98.1% ± 1.3%	98.4% ± 1.2%	0.99 ± 0.0001	0.97 ± 0.025

Comparing Table 10 with Table 6, the proposed VMD-based CNN outperforms the CWT-based CNN in terms of the accuracy, sensitivity, specificity, AUC and QWK with almost 7% improvement across the different sleep-stages. This may indicate that VMD is more robust as compared to CWT to artifacts and noise that may compromise the discriminative features of PD-MCI.

We have also observed that the accuracy of the proposed VMD-based CNN model did not significantly change across the different sleep stages or at the various brain regions where there is almost a 0.8% deviation between the best accuracy at N3 (F3) and the least accuracy at REM (C4). Although the proposed model has the highest sensitivity of 99.5% for detecting PD-MCI from N3 EEG data at F4 channel, there was no significant deterioration in the sensitivity of the model applied on other sleep-stages or brain regions. In addition, the QWK for the proposed VM-based model was almost comparable to the accuracy metric across the sleep stages and brain regions which indicate that the predictions of the model did not take by chance and the high agreement between the predictions and the ground truth annotations (i.e., PD-MCI or PD-NC) are based on reliable and unique features of the disease.

**Fig 5:**
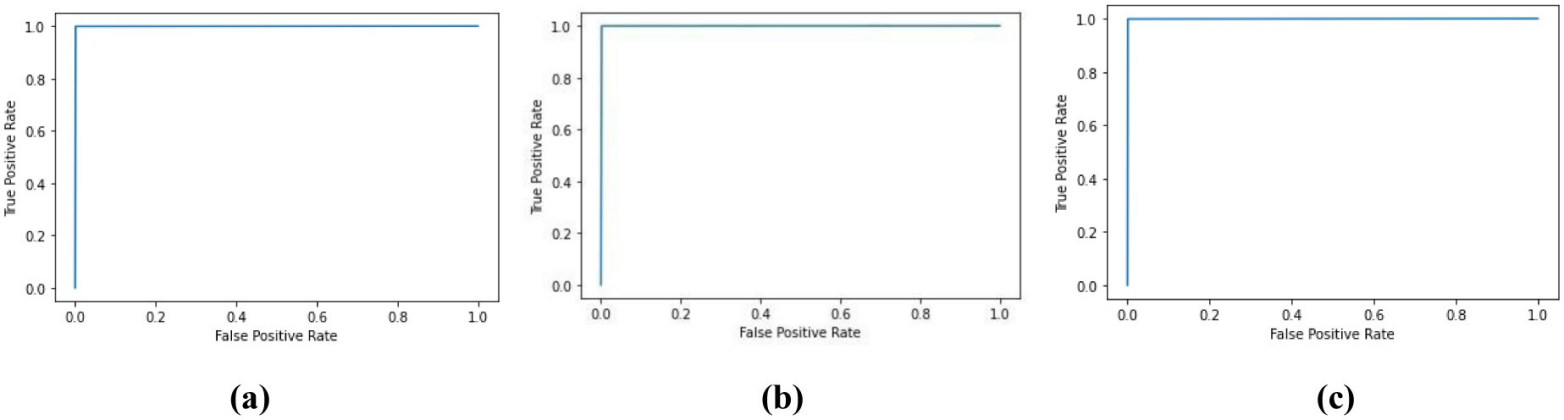
ROC of the proposed classifier applied on (a) N2 (C3) (b) N3 (F3) (c) REM (C3).

Fig 5 shows the ROC of the proposed VMD-based CNN across the three sleep-stages indicating the superior separability of the classifier with an almost 100% true positive rate at a very low false positive rate (~0%).

The confusion matrix for the relatively worst-performing VMD-based CNN model when applied on N2 (C3) data is provided in Table 11 as follows.

**Table 11:** Confusion matrix of the proposed CWT-based CNN on N2 (C3).

	NC	MCI
**NC**	39,654	523
**MCI**	536	35,177

Comparing the confusion matrix for the VMD-based CNN with that of the CWT-based CNN, the proposed VMD-based CNN classifier outperforms and surpasses the CWT-based CNN model in the ability to differentiate MCI from NC as well as exhibiting relatively lower missed detection and false alarm rates.

### 5.3 Computational-time analysis

In addition to evaluating the performance of the proposed frameworks, we have measured the time taken by the proposed approaches to execute the TFR representation operation and CNN classification on a desktop computer with the following specifications: Intel(R) Xeon(R) Gold 5222 CPU@ 3.8 GHz, RAM of 80GB, NVIDIA Quadro P4000 GPU, and Graphics Memory @ 48,858 MB. Table 12 shows the computational-time for both the CWT-based and VMD-based deep-learning frameworks in order to classify 50 segments representing 50 seconds of the patient brain behavior into PD-NC or PD-MCI.

**Table 12:** Comparison of the computational-time for both the CWT-based and VMD-based CNN frameworks.

Framework	Operation	Time-delay
**CWT-based CNN**	**CWT**	18.03 s
	**CNN**	3.54 s
**VMD-based CNN**	**VMD**	6.4 hrs
	**CNN**	5.4 s

From Table 12, it is clear that there is a slight difference between the time-delay incurred by the two CNN models applied on the CWT and VMD data respectively (i.e., ~2s). However, the VMD operation is extensively complex and required almost 6.4hrs to execute while the CWT application took slightly over 18 seconds. Hence, the VMD-based CNN framework may offer a superior performance over the CWT-based CNN, however this is achieved at the cost of an additional computational-time overhead (~6.4hrs). This may burden the real-time realization and application of the VMD-based framework.

### 5.4 Feature visualization and interpretation

**Fig 6:**
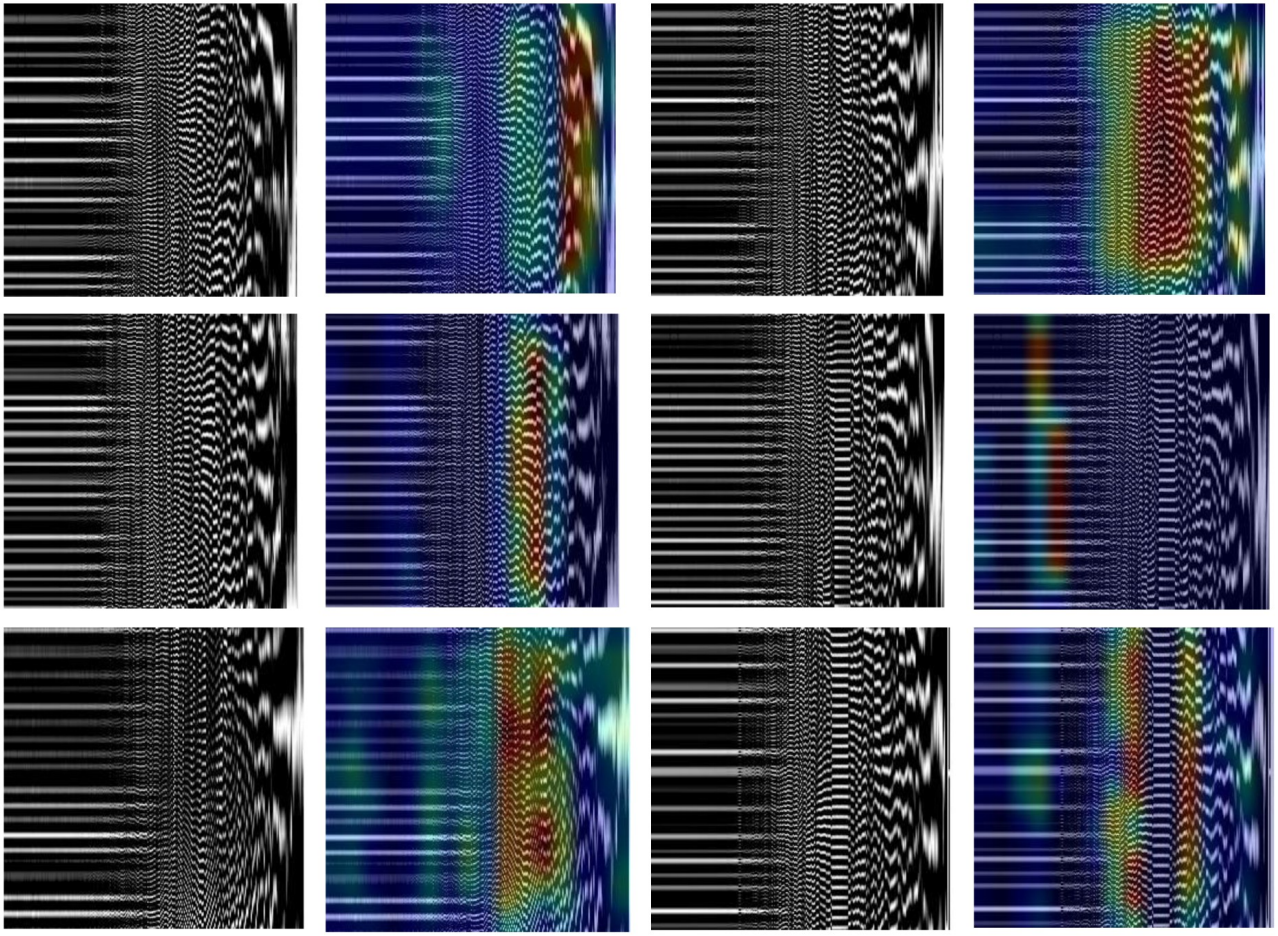
VMD gray-scale images and their corresponding heat maps where the rows from top to bottom represent the N2 (C3), N3 (F3) and REM (C3) respectively. Also, the first and last two columns represent the VMD images for PD-MCI and PD-NC respectively.

Due to the significant improvement in performance for the proposed VMD-based CNN with respect to the CWT-based CNN, we have focused in this section to visualize, highlight and interpret the significant features in the VMD images that triggered the highly accurate prediction of PD-MCI or PD-NC. Fig 6 shows the VMD images for PD-MCI and PD-NC across N2 (C3), N3 (F3) and REM (C3) and the corresponding generated heat maps when the Grad-CAM [79] was used. The Grad-CAM method calculates the averages of the gradients of the score of a certain class (i.e., PD-NC or PD-MCI) with respect to the feature maps of the last convolutional layer of the proposed VMD-based CNN. This operation can be defined as follows:

$$\alpha_{k}{}^{c}=\frac{1}{Z_{k}}\;\sum_{i}\sum_{j}\frac{dy^{c}}{dA_{ij}^{k}}$$ (3)

where *y*^*c*^ is the score value generated by the model at the node/neuron designated for the class ‘*c*’ prior to the application of the SoftMax function, *Z*_*k*_ is the size of the *k*^*th*^ feature map (i.e., pixel count of the feature map), and $A_{ij}^{k}$ are the pixel values of the *k*^*th*^ feature map. Finally, the class discriminative maps (which are represented using heat maps) for a certain VMD image are then calculated as follows:

$$Grad-CAM_{c}=ReLU\left(\sum_{k}\alpha_{k}^{c}*A_{k}\right)$$ (4)

where *ReLU* is the rectified linear unit.

From Fig 6, we have observed that across the three sleep-stages, the proposed VMD-based CNN model was able to predict PD-MCI based on changes in the mid-frequency (for N3 and REM) as well as high-frequency (for N2) VMD components (i.e., marked in red and yellow). Although the patterns may not be easily interpretable by the human eye, this shows that those components carry significant discriminative features of the disease. Further, PD-NC was distinguishable from PD-MCI based on changes attributed to low-frequency (for N3) as well as mid-frequency (for N2 and REM) VMD components.

### 5.5 Comparative study of the proposed work and the-state-of-the-art methods

Although a comparative analysis of the proposed CNN models with traditional machine learning approaches including but not limited to SVM, KNN and multilayer perceptron (MLP) would provide a justification for the selection of deep-learning in exploiting the sleep EEG for patients with PD rather than standard machine learning, the use of machine learning techniques will be challenging as the techniques require one-dimensional inputs (e.g., sleep EEG time-series) rather than a two-dimensional input (e.g., CWT and VMD samples/images). Further, we have previously experimented the use of standard machine and deep-learning techniques directly on the time-representation of sleep EEG and the classification accuracy was minimal. This may be due to the challenges related to the inability to extract unique and discriminative features of the disease from instantaneous time-series data that may be prone to noise and artifacts. In addition, the past studies [6, 7, 15–19] have focused on exploiting certain frequency bands of the EEG including *δ*, *θ*, *β* and γ frequency bands in order to classify subjects into patients with PD and HC. That motivated the use of two-dimensional TFR components of the sleep EEG in this study as features of PD with MCI may be attributed to changes in both the time and frequency domains.

Despite not using the-state-of-the-art pre-trained models including VGG-16, DenseNet, ResNet-50, MobileNet, etc. in our analysis, we have achieved a significantly high accuracy using the proposed CNN models up to 95.9% and 99.8% when the CWT and VMD data were used. In this study, we proposed novel CNN models with less number of layers and low computational complexity with respect to most of the-state-of-the- art pre-trained models including ResNet-50, MobileNet, DenseNet among others. The number of layers used in the CWT-based CNN was 14 layers while the number of layers used in the VMD-based CNN was 19 layers as compared to 50 layers used in ResNet-50 and 203 layers used in DenseNet and that may eventually minimize model overfitting.

In addition, the proposed models were adapted to the size of the input images representing the 176 ×176 or 146 ×146 CWT samples and the 256 × 256 VMD samples without losing significant details due to scaling the images (i.e., oversampling or undersampling the images) to fit into the input layer of the models. For pre-trained models, images need to be scaled to a fixed size such as 224 × 224 as in VGG-16 and ResNet-50 which may influence the quality of the images, compromise the amount of detail and features related to the disease in the CWT/VMD samples and eventually limit the ability of the models to identify distinctive and reliable features of the disease.

Since to the best of our knowledge this is considered the first deep-learning framework that has been developed to exploit the sleep EEG for patients with PD to identify the features of MCI and NC, it is challenging to conduct an objective comparison between the proposed work and other related works that have been limited to the use of deep-learning for PD detection, staging and biomarkers identification based upon awake EEG. Further, the dataset that has been used in this study was recorded for PD patients during sleep while other datasets are mostly related to resting-state EEG. Finally, we were able to provide a holistic comparative study of the proposed work and the related state-of-the-art deep-learning methods applied on the TFR of the awake EEG for patients with PD as shown in Table 13.

**Table 13:** Comparison of the proposed approach and the-state-of-the-art architectures for PD detection.

Approach	Dataset	TFR	MDL	Classification	Accuracy
Khare *et al*., 2021 [58]	UC San Diego Resting State EEG for 15 PD and 16 HC	TQWT	LSSVM	HC Vs. PD (OFF Medication) HC Vs. PD (ON Medication)	96.13% 97.65%
Khare *et al*., 2021 [59]	1. UC San Diego Resting State EEG for 15 PD and 16 HC 2. UKM Medical Center EEG for 20 PD and 20 HC	Smoothed Pseudo Wigner Ville Distribution	7-Layer CNN	HC Vs. PD (OFF Medication) HC Vs. PD (ON Medication)	99.7% 100%
Loh *et al*., 2021 [60]	UC San Diego Resting State EEG for 15 PD and 16 HC	Gabor Transform	5-Layer CNN	HC Vs. PD (ON, OFF Medication)	99.46%
Shaban *et al*. [63]	UC San Diego Resting State EEG for 15 PD and 16 HC	CWT	17-Layer CNN	HC Vs. PD (OFF Medication) PD (OFF Medication) Vs. PD (ON Medication) HC Vs. PD (ON, OFF Medication)	99.9% 99.8% 99.6%
Khare *et al*. [65]	UC San Diego Resting State EEG for 15 PD and 16 HC	AOVMD	AOELM	HC Vs. PD (OFF Medication) HC Vs. PD (ON Medication)	98.9% 98.6%
Chawla *et al*. [66]	1. UC San Diego Resting State EEG for 15 PD and 16 HC 2. UKM Medical Center EEG for 20 PD and 20 HC	FAWT	KNN	HC Vs PD	99% 95.9%
**Proposed Work**	UAB Sleep EEG for 16 PD-MCI and 20 PD-NC	CWT VMD	14-Layer CNN 19-Layer CNN	PD-MCI Vs. PD-NC	Up to 95.9% Up to 99.8%

While it is not relevant to compare our proposed method with the current literature due to the differences in the objective and the datasets used, the proposed CWT-based and VMD-based deep-learning frameworks still provide a comparable high detection accuracy for PD-MCI with respect to the related work.

## 6. Discussion

PD is the second most common neurodegenerative disease in the United States caused by the degeneration of neurons in the substantia nigra of the brain. It is expected that the number of patients who suffer from the disease will rise to 1.2 million by 2030 with almost 90,000 individuals being diagnosed with PD each year according to the Parkinson’s foundation [3]. By the time of onset of motor symptoms and diagnosis, almost 60%-80% of the dopaminergic nerve cells are damaged in the substantia nigra of the brain [1]. At that point, the clinical diagnosis is mainly based on the observation of the abnormalities in the motor system and is considered subjective and prone to human error [3]. Accordingly, an early diagnosis and a timely initiation of efficient therapeutic and neuroprotective treatments are crucial to improve the quality of life for the patients and potentially slow down the progress of the disease. Although EEG is not currently used for the clinical diagnosis of PD, several studies have shown its promise for identifying unique and discriminative features for PD and cognitive dysfunction complications [6–11, 15–19]. In addition, age, sex, behavioral state (wake vs sleep), medication regime, time of recording, EEG electrode position capturing the neural activity within a certain region of the brain, sampling rate, and the artifacts or noise caused by patient’s motion or REM are all important factors that need to be considered when conducting a clinical evaluation of PD based on EEG.

Other studies have introduced machine and deep-learning approaches to differentiate PD from HC, support the staging and biomarker identification of the disease based upon EEG, MRI, speech tests, handwriting tests and sensory data [30–72]. In this study, we have proposed two TFR based deep-learning frameworks where the sleep-stage (i.e., N2, N3 and REM) EEG signals of overnight recorded EEG for PD patients with NC and MCI were extracted while CWT or VMD transformation was applied on the segments and further two novel CNN models were developed to classify the CWT or VMD segments into PD-NC or PD-MCI. The proposed CWT-CNN provided an accuracy of 92% to 96% across the three sleep-stages and at four selected electrodes. In addition, the proposed VMD-CNN offered a significantly high accuracy of 99.2% to 99.8% with a corresponding QWK of 0.97 to 0.98 proving the reliability and preciseness of the models.

Further, it was also observed that the performance of the proposed CWT-based CNN models at the frontal regions are relatively higher than that at the central regions. There is almost 3.8% improvement in the performance of the model when applied on the N3 EEG data at the frontal channel F4 over the same model when applied on N2 EEG data at the central channel C3. However, there is no significant improvement of the application of the VMD-based CNN on the frontal regions of the brain with respect to the central regions. It is also worth mentioning that our analysis was focused on studying certain brain regions for each sleep-stage due to the relevance of the features at those locations for the discrimination of PD-MCI from PD-NC. It has also been shown that PD-MCI was attributed with changes in the mid- or high-frequency VMD components while PD-NC was characterized by variations in the low- and mid-VMD components as determined by the Grad-CAM method.

The proposed frameworks serve as accurate as well as reliable computer aided-diagnostic tools for neurologists to perform pre-screening of MCI in patients with PD in order to monitor the progression of the disease to MCI based upon EEG signals recorded during each sleep stage. This will also reduce the total cost of unnecessary cognitive testing for PD patients who do not have MCI. In addition, this tool can also be used to minimize the number of failed clinical trials identifying PD patients with MCI conducted by sleep medicine scientists.

Although the proposed frameworks provided promising results in differentiating PD-MCI from PD-NC, it has the following limitations:

1. The size of the dataset that the models have been trained and validated on is limited. In addition, the models have not been tested on real-time clinical data which is always a challenge facing deep-learning studies in the neuroscience field. However, our future plans will include introducing a graphical user interface based software that implements the deep-learning frameworks we have proposed and the software can be used by clinicians to test on new EEG data for patients with PD and compare with their cognitive testing results.
2. In this study, we have not directly applied deep-learning on each IMF of a total of 256 IMFs or subsets of IMFs generated by the VMD method to investigate the significance of each individual IMF in the prediction outcome. This would have offered further insights into the vital and significant frequency sub-bands that are useful in differentiating PD with MCI from PD with NC. However, in this study, we were able to highlight the range of IMFs that significantly influences the prediction of MCI using the Grad-CAM method. We have concluded that across most of the VMD samples/images related to the three sleep-stages, the mid and high frequency VMD components present unique and significant features for MCI detection.
3. Although, we were able to identify samples based upon the sleep EEG for PD subjects with and without MCI that have distinctive and discriminative features of the disease using a simple and fast continuous wavelet transform and can be further classified with the proposed CNN at a high accuracy (i.e., above 92%), the use of more efficient techniques including Discrete Wavelet Transform (DWT), Wavelet Packet Transform (WPT), Gabor and Margenau–Hill transforms will be further investigated to better understand unique features of PD with MCI and dementia as well as early or prodromal PD using a larger dataset to be collected for patients during sleep.
4. Although the proposed CWT-based CNN showed a significant improvement in classifying subjects into PD-MCI and PD-NC when applied on the N3 segments of the sleep-EEG, which is consistent with the findings of a recent study that demonstrated the correlation between the N3 *δ* waves and cognition in PD [80], it would be useful to develop a proposed framework that exhibit the ability to automatically segment N3 time-frames from the EEG time-series and further classify the signals into MCI and NC.
5. Although the proposed VMD-based CNN provides a superior performance with respect to the CWT-CNN framework, the computational-time required to execute the VMD transformation of 50 segments exceeds 6 hours which is not appropriate for the real-time application and testing of the framework. In the future, we will address the optimization of the VMD method and identify techniques that minimize the size of the input to the VMD method.
6. Our proposed approach provides a precise prediction of the presence of MCI in PD patients who have already developed MCI, However, it would be interesting if the proposed approach predicts the risk of the progression to MCI. This can only be realized by having access to sufficient sleep EEG data recorded for patients who have previously been with NC but later progressed to MCI and further using the deep-learning models to classify PD subjects into NC who have not progressed to MCI and patients who have developed MCI. This would provide outstanding support for specialists to recognize the risk for each patient and take proactive steps to slow down the progression of the disease using effective and efficient therapeutic treatments.

## Supporting information

S1 Fig This is an example for the CWT of N2 (C3) EEG data for a patient with NC. (JPG)

S2 Fig This is an example for the CWT of N2 (C3) EEG data for a patient with MCI. (JPG)

S3 Fig This is an example for the VMD of N2 (C3) EEG data for a patient with NC. (JPG)

S4 Fig This is an example for the VMD of N2 (C3) EEG data for a patient with MCI. (JPG)
