# Supplementary figures and images for: Deep-learning detection of mild cognitive impairment from sleep electroencephalography for patients with Parkinson’s disease
Source: PLoS One. 2023 Aug 3;18(8):e0286506. doi: 10.1371/journal.pone.0286506 (PMC10399849; doi:10.1371/journal.pone.0286506)

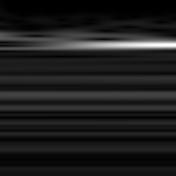

Supplement: S1 Fig — (JPG) [file pone.0286506.s001.jpg]

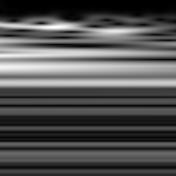

Supplement: S2 Fig — (JPG) [file pone.0286506.s002.jpg]

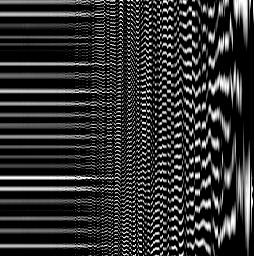

Supplement: S3 Fig — (JPG) [file pone.0286506.s003.jpg]

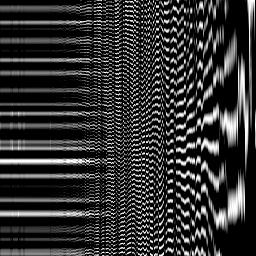

Supplement: S4 Fig — (JPG) [file pone.0286506.s004.jpg]
